# Perceptions of the possible health and economic impacts of Seattle’s sugary beverage tax

**DOI:** 10.1186/s12889-019-7133-2

**Published:** 2019-07-09

**Authors:** Vanessa M. Oddo, James Krieger, Melissa Knox, Brian E. Saelens, Nadine Chan, Lina Pinero Walkinshaw, Mary Podrabsky, Jessica C. Jones-Smith

**Affiliations:** 1 Department of Health Services and Center for Public Health Nutrition, University of Washington School of Public Health, 330 Raitt Hall, Seattle, WA 98195 USA; 2 Healthy Food America and Departments of Medicine and Health Services, University of Washington, Box 22260, Seattle, WA 98122 USA; 3 Department of Economics, University of Washington, 305 Savery Hall, Seattle, WA 98195 USA; 4 Seattle Children’s Research Institute and Department of Pediatrics, University of Washington School of Medicine, 2001 8th Ave, Suite 400, Seattle, WA 98121 USA; 5 Public Health - Seattle and King County and Department of Epidemiology, University of Washington School of Public Health, 401 5th Ave, Suite 1300, Seattle, WA 98104 USA; 6 Departments of Health Services and Epidemiology and Center for Public Health Nutrition, University of Washington School of Public Health, 305-G Raitt Hall, Seattle, WA 98195 USA

**Keywords:** Sugary beverages, Sugary beverage tax, Seattle, Tax support

## Abstract

**Background:**

Taxes on sugary beverages are an emerging strategy to improve health by reducing consumption and raising revenues to support community wellbeing. However, taxes may have unintended consequences, and perceptions of these consequences may affect attitudes towards this policy.

**Methods:**

In June 2017, the Seattle City Council passed an ordinance imposing a tax on sugary beverages, effective January 1, 2018. Between October and December 2017, we recruited 851 adults in Seattle to complete a survey (telephone or online) about support for the tax and their perceptions of tax-related health and economic impacts. We first analyzed data for the full sample. We then tested for differences in participants’ responses by household income level (< 260% Federal Poverty Level [FPL], ≥ 260% FPL) and across race/ethnicities using chi-square tests. Analyses used population weights and adjusted for multiple comparisons, using the Holm-Bonferroni Sequential Correction (*p* < 0.01).

**Results:**

A majority of participants supported the sugary beverage tax (59%; 95% Confidence Interval [CI]: 55, 63%) and believed that the tax would improve public health (56%; CI: 52, 60%). Most participants believed that the tax would not negatively affect small businesses (52%; CI: 48, 56%) nor result in job loss (66%; CI: 62, 70%). Most participants also perceived that the tax would not negatively impact their own finances (79%; CI: 75, 82%). However, fewer lower-income (48%; CI: 42, 53%), versus higher-income participants (61%; CI: 55, 66%), perceived that the tax would improve public health, would not result in job loss (lower-income: 58%; CI: 53, 64%; higher-income: 71%; CI: 66, 75%) and would not negatively affect their own finances (lower-income: 68%; CI: 62, 73%; higher-income: 85%; CI: 81, 88%). Compared to non-Hispanic Whites, (82%; CI: 79, 86%), a smaller proportion of non-Hispanic Blacks (63%; 95% CI: 48, 75%), and Hispanics (67%; 95% CI: 51, 79%), perceived that the tax would have negative consequences for their own family finances.

**Conclusions:**

A majority of respondents supported the sugary beverage tax in Seattle. Lower-income participants were more concerned about potential financial consequences. Further evaluation of the extent to which unintended consequences occur is needed.

**Electronic supplementary material:**

The online version of this article (10.1186/s12889-019-7133-2) contains supplementary material, which is available to authorized users.

## Background

There is growing momentum for sugary beverage taxes as a promising strategy to reduce obesity and chronic disease, by reducing consumption and generating revenues to improve the health and wellbeing of communities disproportionately affected by the marketing and adverse health consequences of sugary beverages [1–3]. Seven U.S. municipalities have implemented taxes: Philadelphia, Pennsylvania; Boulder, Colorado; Seattle, Washington; and Berkeley, San Francisco, Oakland, and Albany, California. However, taxes may have unintended consequences, and perceptions of these consequences may affect attitudes towards this policy.

Evidence from Berkeley [4] and early results from Philadelphia [5] find that sugary beverage taxes decrease sugary beverage consumption. Taxes are generating more than $125 million annually and this revenue is being invested in low-income communities to promote health and support education. However, concerns about unintended consequences [6–9] have also emerged, and were prominent during the public debate on the sugary beverage tax in Seattle [10]. In Seattle, both community organizations and the beverage industry noted that the tax could have regressive fiscal impacts on people with low incomes. Other stakeholders, called out the potential loss of small business revenues and jobs in the beverage and food retail sectors, and suggested that revenue loss might disproportionately affect minority-owned businesses. The City Council took these concerns seriously and included provisions in the final legislation to address them [11]. Foremost, the Ordinance notes that the intent of the legislation is to raise revenues to expand access to healthy and affordable food to low-income communities and to reduce disparities in education, health and social inequities. In turn, the disbursement of the revenue is being guided by a diverse Community Advisory Board that will help ensure that the tax revenues are allocated consistent with the legislative intent. In addition, the Ordinance exempts sugary beverages made by very small manufacturers and authorizes funding for job placement programs for those workers adversely affected by the tax. However, among Seattle residents, it is unclear how widespread concerns are about the potential negative impacts on employment, loss of sales revenues, and/or regressive fiscal impacts on people with low-income and people of color.

Moreover, a broader body of literature suggests that support for sugary beverage taxes may differ by demographic characteristics [7, 9, 12–14]. For example, a few cross-sectional studies have found that lower (versus higher) educational attainment is associated with lower levels of support for sugary beverage taxes [9, 13] and lower odds of perceiving that the tax would improve public health [7]. Prior studies have also reported higher levels of support for sugary beverage taxes among younger-aged participants (versus older-aged) [9, 12, 14], and unexpectedly, higher levels of support among individuals who identified with a race/ethnicity other than White (i.e. Black, Hispanic, Asian, or Multiracial) [13], and those with lower (versus higher) levels of household income [12].

The objective of this study was to examine adults’ support for Seattle’s sugary beverage tax, after tax adoption and prior to implementation, as well as their perceptions of the possible health and economic impacts of the tax. We also investigated whether perceptions of the tax and tax impacts differed by income level and race/ethnicity. A better understanding of public perceptions of sugary beverage taxes can assist policymakers, scientists, and communities in mitigating potential concerns as they seek to adopt or implement sugary beverage taxes. For example, tax policy can include provisions for revenue dedication to offset any regressive tax effects or support for small businesses concerned about revenue losses. Communications about the tax can also address concerns.

## Methods

### Survey design

On June 6, 2017, the Seattle City Council passed Ordinance 125324 imposing a tax on distributing sugary beverages in Seattle. Tax implementation began on January 1, 2018. In Seattle, large distributors now pay a 1.75 cents per ounce tax on sugary beverages. Taxed beverages include drinks that have added sugar. The tax does not include diet beverages, 100% fruit juices, or milk products. The tax was intended to address equity issues, as revenues from the tax are being invested in programs that increase access to healthy and affordable food, expand early education for pre-school aged children, and help high school graduates enter college [11]. We designed a survey to investigate Seattle residents’ perceptions about the tax itself and their views on the potential positive and negative health and economic impacts of the tax. These analyses focused on survey questions that addressed perceptions about: (1) the tax itself (5 items); (2) the health and economic impacts of the tax (6 items); and (3) demographic characteristics (12 items).

### Data collection

Data were collected by a survey research firm, Ironwood Insights Group, LLC, between October and December 2017, using a mixed-mode (telephone and online) survey. The telephone survey was conducted by trained interviewers and standard data quality assurance checks were employed (e.g. live monitoring of approximately 10% of all calls over the survey period). Participants were contacted up to 6 times via telephone and 2 times on average. They were not compensated for participation.

Phone survey participants were selected using a stratified random sampling approach, further described below, sampling from databases of all working landline and cell phone numbers for Seattle. Participants who completed the survey online were selected from several existing panels comprised of a large sample of individuals who either completed online surveys or opted in to participate in online surveys in the past. All Seattle residents age 18 and older were eligible for inclusion. Those refusing to answer the screener questions on income and race/ethnicity or who did not speak or read English or Spanish or read Vietnamese were ineligible.

We designed the survey to be able to test whether opinions about the tax were different for lower-income versus higher-income populations. Lower-income was defined as < 260% of the Federal Poverty Line (FPL). We estimated that in order to detect a 10 percentage point difference in tax approval between higher- and lower-income participants, with a power of 80% and a 5% probability of Type I error, assuming a 60% tax approval rating, we would need a sample of 356 participants per income group. We successfully recruited 456 higher-income participants and 395 lower-income participants. We also aimed to recruit a sample that had the same race/ethnic distribution as the population of Seattle, based on the 5-year American Community Survey (ACS) sample (2012–2016).

We recruited a total of 851 participants (46% completed by phone and 54% completed online). Similar to response rates in national-level random digit dial surveys [15, 16] and a recent evaluation of the sugary beverage tax in Philadelphia, [5] our survey had a response rate of 3.6% for participants contacted via landline and 6.7% for participants contacted via cellphone, estimated using the American Association for Public Opinion Research Response Rate Number 4 (see Additional file 1: Table S1) [17]. We were not able to estimate a response rate for our online sample; however, the demographic characteristics were mostly similar by mode (telephone and online) across income and race/ethnicity (Additional file 1: Table S2).

The phone and web versions of the survey were offered in English and Spanish and we also offered the web version of the survey in Vietnamese. The University of Washington School of Public Health Institutional Review Board determined that this study was exempt.

### Description of key variables

#### Primary independent variables

Participants were categorized as having incomes below <260% FPL or ≥ 260% FPL based on their self-reported total annual household income and given household size, defined using the annual federal poverty guideline (see Additional file 3 for survey questions) [18]. We created mutually exclusive categories for race/ethnicity, based on self-reported answers to two separate questions about race and ethnicity. Based on responses, individuals were categorized as follows: non-Hispanic White, non-Hispanic Black, non-Hispanic Asian, non-Hispanics of “other” race, or Hispanic. Native Hawaiian or Other Pacific Islanders, American Indian and Alaska Natives, and individuals who reported two or more races, were categorized as non-Hispanic of an “other” race because Native Hawaiian or Other Pacific Islanders and American Indian and Alaska Natives each make up a very small segment of the population in Seattle when categorized separately.

Additionally, participants were asked to indicate which of five categories reflected their education (some high school, completed high school, some college or university, completed graduate or professional degree). Participants were also asked to indicate their gender (male, female, self-identify), their age (18–30 years old, 31–40 years old, 41–50 years old, 51–64 years old, ≥ 65 years), annual household income (<$30,000, $30,000–$59,999, $60,000–$89,999, $90,000–$120,000, >$120,000), political affiliation (Democrat, Republican, Independent, Other), and whether they had heard of the tax prior to participating in the survey (yes, no, don’t know). In addition, participants were asked about their consumption of sugary beverages during the prior 30 days, using a modified version of the NHANES Dietary Screener Questionnaire (none or < 1 per week, 1 per week, 2–6 per week, 1 per day, ≥ 2 day, don’t know) [19].

#### Primary dependent variables

The primary dependent variables included participants’ opinion about the tax and participants’ perceptions regarding the potential impact of the tax on: child well being, public health, cross-border shopping, small businesses, the Seattle economy, job loss, family finances, tax effects on people with low-income and people of color, and autonomy to choose what beverages one drinks. Questions were queried as four-category variables and there was also an option to report “don’t know” or “refused” (see Additional file 3). In this survey, we described the tax itself (e.g. 1.75 cents per oz), explained what the tax revenue would be used for in Seattle (e.g. increased access to healthier food) and then asked participants’ opinion about the tax itself using a four-category Likert scale, with response options of strongly approve, somewhat approve, somewhat disapprove, and strongly disapprove. Then, to assess participants’ perceptions about the health and economic impacts of the tax, each participant was read two statements and asked to indicate if the first statement was much or somewhat closer or the second statement was much or somewhat closer to her/his belief. For example, participants were asked whether the statement *I will travel to another city to buy sugary drinks so I don’t have to pay the tax* was somewhat or much closer to their own view as compared to the statement *I will not travel to another city to buy sugary drinks because of the tax*. For simplifying the reporting of the results, we collapsed the responses from four-category variables to two-category variables, since preliminary analyses indicated that the direction of the associations and statistical significance were similar when using a two- or four-category variable. “Strongly” and “somewhat” agree were collapsed into “agree”, “strongly” and “somewhat” disagree were collapsed into “disagree”. Similarly, “somewhat” and “much” closer for the matched pair statements were collapsed to capture respondents’ agreement with that statement. In our analyses, we also report the “don’t know” responses, but “refusals” were coded as missing values.

In addition to examining individual survey items, we created a score to summarize overall perceptions of perceived health and economic impacts of the tax (henceforth referred to as the tax impacts score). The tax impacts score was comprised of the nine questions related perceived tax impacts on: child well being, public health, cross-border shopping, small businesses, the Seattle economy, job loss, family finances, impacts of the tax on low-income people and people of color, and autonomy over beverage choice. A participant received a 1 if they responded that the impact of the tax would be positive/beneficial (e.g. tax will improve public health), a 0 if they responded that they “don’t know”, and a − 1 if they responded that the impact of the tax would be negative/detrimental (e.g. tax will not improve public health) (score range − 9 to + 9). A higher score indicated that the tax was perceived to have more positive impacts on health and economic factors.

### Statistical analysis

#### Analyses of specific questions

We first estimated participants’ perceptions of the impacts of the tax for the entire sample. Based on prior evidence that documents differences in consumption by race/ethnicity and income [20, 21, 31, 32], we hypothesized that perceptions about the tax would also vary by these factors. Therefore, we then used chi-square tests to test for differences in participants’ response to survey questions by high- versus low-income (3 × 2 chi-square) and across all race/ethnicities (3 × 5 chi-square)(e.g. were there any statistically significant differences in the proportion of respondents who reported “agree”, “disagree”, or “don’t know”, comparing all race/ethnicities to each other). We did not test for statistical differences between each racial/ethnic group in order to avoid excessive statistical testing and because we were not powered to do so. In supplemental analyses, we also describe tax support by gender, age, education level, political affiliation, participants’ prior knowledge of the tax (i.e. had they ever heard of the tax) and consumption (none or < 1 per-week, ≥ 1 per-week), but we do not test for statistical differences between each group to avoid excessive statistical testing.

#### Analyses of tax impact score

In addition, we aimed to better understand the association between demographic characteristics and overall perceptions of the tax, as measured the tax impact score, on a continuum. We first used unadjusted linear regression models, with robust standard errors, to estimate whether income was associated with the tax impacts score. In a second, separate model, we estimated whether race/ethnicity was associated with the tax impacts score. We then used linear regression models to further explore the association between income and race/ethnicity and the tax impacts score, in one model that mutually adjusted for income (<260% FPL, ≥ 260% FPL) and race/ethnicity, while also controlling for education, sex, age, and political affiliation.

All results presented are based on analyses using survey weights, constructed using the raking method, an iterative proportional weight (see Additional file 2) [22]; weights adjusted results to the known City of Seattle population totals (as determined by the 5-year ACS) for race/ethnicity, gender, age, and annual median household income. All statistical analyses were performed using Stata 15.1 (StataCorp LP, College Station, Texas). Both chi-square and linear regression results were adjusted for multiple comparisons using the Holm-Bonferroni Sequential Correction, in order to determine statistical significance (*p* < 0.01 in these analyses) [23]. First, results for both chi-square and the linear regressions were each ordered from the smallest *p*-value to the largest *p*-value. Second, the second smallest *p*-value is corrected with a Bonferroni approach ([number of tests − order of test + 1] × *p*-value). The correction procedure stops when the first non-significant test is obtained.

## Results

The weighted sample characteristics are displayed in Table 1 (see Additional file 1: Table S3 for unweighted sample characteristics and comparison of sample to ACS). The population was 66% non-Hispanic White, 7.0% non-Hispanic Black/African American, 14% non-Hispanic Asian, 6.7% non-Hispanic of another race, and 6.6% Hispanic. Approximately, 70% of the weighted sample completed college or had a graduate degree and 40% of the sample was lower-income (< 260% FPL). About two-third of the participants had heard of the tax prior to our survey. In Seattle, 55% of participants reported consuming a sugary beverage at least once per-week and 17% of participants consumed a sugary beverage at least once per-day.

**Table 1:** Selected Characteristics of survey participants in Seattle (*N* = 851)^a,b^

	N	Weighted %
Gender		
Male	349	50%
Female	499	50%
Race/Ethnicity		
Non-Hispanic white	588	66%
Non-Hispanic Black/African American	60	7.0%
Non-Hispanic Asian	66	14%
Non-Hispanic Other^c^	78	6.7%
Hispanic	56	6.6%
Age		
18–30 years old	133	19%
31–40 years old	152	22%
41–50 years old	136	21%
51–64 years old	167	24%
≥65+ years old	250	14%
Education		
Some high school	24	2.1%
Completed high school	79	7.9%
Some college or vocational training	199	21%
Completed college or university	294	38%
Completed graduate or professional degree	241	31%
Income Level Relative to FPL		
Lower Income: < 260% FPL	395	37%
Higher Income: ≥ 260% FPL	456	63%
Household Level Income		
<$30,000	242	21%
$30,000-59,999	213	21%
60,000-89,999	137	22%
90,000-120,000	92	15%
>$120,000	126	22%
Political Affiliation		
Democrat	462	57%
Independent	236	32%
Republican	71	10%
Other	13	2.0%
Don’t know	45	1.0%
Participant Heard of Tax		
No	198	24%
Yes	634	73%
Don’t know	19	2.2%
Consumption of Sugary Beverages		
None or < 1 per week	406	44%
1 per week	132	16%
2–6 per week	169	22%
1 per day	72	9.0%
2 + day	62	8.0%
Don’t know	10	1.1%

*FPL* federal poverty line

^a^N is unweighted to show actual sample size whereas percentages (%) are based on weighted to the ACS (2012–2016). Therefore, the percentages displayed will be different from the number you get by dividing the total N by the cell-specific N. Percentages are rounded to two significant digits

^b^Missing data: gender (*n* = 3); ethnicity (*n* = 3 [individuals who responded “don’t know”]); age (*n* = 13), education (*n* = 14), household income (*n* = 41); political affiliation (*n* = 24)

^c^Native Hawaiian or Other Pacific Islanders, American Indian and Alaska Natives, and those reporting two or more races are categorized as non-Hispanic Other

### Perceived impacts of the tax in Seattle

In the overall sample (*N* = 851), a majority of participants supported the sugary beverage tax in Seattle (59%; 95% Confidence Interval [CI]: 55, 63%) and correspondingly, believed that the tax would help improve health (Table 2). In particular, 59% (95% CI: 55, 63%) of participants perceived that the sugary beverage tax would improve the health and wellbeing of children, and 56% (95% CI: 52, 60%) believed the tax would improve the public’s health more generally. Most participants believed that the tax would not negatively affect small businesses in Seattle (52%; 95% CI: 48, 56%) nor result in job loss (66%; 95% CI: 62, 70%). A large majority perceived that the tax would not negatively impact their own finances (79%; 95% CI: 75, 82%). About half (48%; 95% CI: 44, 52%) (47%; 95% CI: 44, 51%) believed the tax would positively impact people with low-income and people of color and believed the tax would have a positive effect on Seattle’s economy (47%; 95% CI: 44, 51%); more than 10% of participants responded that they “don’t know” to these questions. Only a quarter (26%; 95% CI: 23, 30%) believed that the tax would limit their choice of beverages.

**Table 2:** Perceived Health and Economic Impacts of Tax in Seattle Overall and by Income Level

	Overall	< 260% FPL	≥ 260% FPL	*p* value
	(*N* = 851)^a,b^	(*N* = 395)^a^	(*N* = 456)^a^	
	% (95% CI)	% (95% CI)	% (95% CI)	
Opinion on Tax^b^				
Approve	59% (55, 63%)	52% (46, 58%)	63% (58, 68%)	0.021
Disapprove	37% (33, 41%)	43% (37, 48%)	33% (28, 38%)	
Don’t know	4.4% (3.0, 6.3%)	5.4% (3.2, 9.0%)	3.8% (2.3, 6.2%)	
Child Well Being^c^				
Tax will improve child health and wellbeing	59% (55, 63%)	53% (47, 59%)	62% (57, 67%)	0.021
Tax will not improve child health and wellbeing	37% (34, 41%)	41% (36, 47%)	35% (30, 40%)	
Don’t know	3.9% (2.6, 5.6%)	5.8% (3.5, 9.5%)	2.8% (1.6, 4.9%)	
Public Health^c^				
Tax will improve public health	56% (52, 60%)	48% (42, 53%)	61% (55, 66%)	<0.001*
Tax will not improve public health	40% (36, 44%)	46% (40, 52%)	37% (32, 42%)	
Don’t know	4.3% (3.0, 6.1%)	5.4% (3.2, 9.0%)	2.9% (1.6, 5.0%)	
Seattle’s Economy^c^				
Tax will have a positive effect on the economy	47% (44, 51%)	44% (38, 50%)	50% (44, 55%)	0.31
Tax will have a negative effect on the economy	35% (32, 39%)	37% (31, 43%)	35% (30, 40%)	
Don’t know	17% (15, 20%)	19% (15, 24%)	16% (13, 20%)	
Cross-Border Shopping^c^				
Participant will not cross-border shop	77% (74, 80%)	72% (67, 77%)	80% (76, 84%)	<0.001*
Participant will cross-border shop	20% (17, 23%)	21% (17, 27%)	19% (15, 24%)	
Don’t know	2.9% (1.8, 4.5%)	6.1% (3.7, 9.9%)	1.0% (0.39, 2.4%)	
Small Businesses^c^				
Tax will not negatively affect small businesses	52% (48, 56%)	47% (42, 53%)	55% (50, 60%)	0.096
Tax will negatively affect small businesses	39% (35, 43%)	42% (36, 47%)	37% (32, 42%)	
Don’t know	9.0% (7.0, 11%)	11% (8.0, 15%)	7.7% (5.4, 11%)	
Job Loss^c^				
Tax will not result in job loss	66% (62, 70%)	58% (53, 64%)	71% (66, 75%)	<0.001*
Tax will result in job loss	23% (20, 26%)	26% (21, 31%)	21% (17, 26%)	
Don’t know	11% (8.7, 13%)	16% (12, 21%)	7.9% (5.6, 11%)	
Family Finances^c^				
Tax will not negatively affect family finances	79% (75, 82%)	68% (62, 73%)	85% (81, 88%)	<0.001*
Tax will negatively affect family finances	18% (15, 21%)	25% (21, 30%)	14% (10, 18%)	
Don’t know	3.6% (2.3, 5.4%)	6.9% (4.4, 11%)	1.6% (0.63, 3.9%)	
Impact on People with Low-income and People of Color^c^				
Tax will positively impact people with low-income/people of color	48% (44, 52%)	44% (38, 49%)	50% (45, 56%)	0.18
Tax will negatively impact people with low-income/people of color	41% (37, 45%)	43% (38, 49%)	40% (35, 45%)	
Don’t know	11% (8.7, 13%)	13% (9.4, 17%)	10% (7.2, 13%)	
Individual Choice^c^				
People will have the choice to drink the beverages they want	71% (67, 74%)	64% (59, 70%)	75% (70, 79%)	0.014
People will not have the choice to drink the beverages they want	26% (23, 30%)	32% (27, 37%)	23% (19, 28%)	
Don’t know	3.0% (1.9, 4.5%)	3.9% (2.1, 7.2%)	2.4% (1.4, 4.2%)	

*CI* confidence interval, *FPL* Federal poverty line

^a^Values are rounded to two significant digits. Missing data: opinion on tax (*n* = 1); cross-border shopping (*n* = 4), small business (*n* = 1); job loss (*n* = 1); impact on low-income people/people of color (*n* = 4); individual choice (*n* = 1)

^b^Responses included: strongly disapprove, somewhat disapprove, somewhat approve, strongly approve, don’t know. These categories are collapsed into: approve, disapprove, don’t know

^c^Participants were read two statements and asked to indicate if the first statement was much closer, the first statement was somewhat closer, the second statement was much closer, or the second statement was somewhat closer. There was also an option to report “don’t know” or “refused” for each of the questions. These categories are collapsed into: the first statement was closer, the second statement was closer or don’t know

^*^Denotes statistically significant difference in participants’ response to all three response categories (agree, disagree, don’t know), comparing lower-income to higher-income participants after considering multiple comparisons, using the Holm-Bonferroni Sequential Correction (*p* < 0.01). Statistical significance estimated using a Chi-squared test

Contrary to the hypothesis that a sugary beverage tax will increase cross-border shopping (i.e. shopping for sugary beverages in nearby areas that are not subject to the tax), 77% (95% CI: 74, 80%) of participants reported that they do not plan to leave Seattle to shop for sugary beverages to avoid paying the tax. Notably, responses were very similar among those participants who live close to the border (defined as within 1 mile of the North or South Seattle border; major bodies of water make up the eastern and western borders of Seattle) as compared to those who did not live close to the border.

### Perceived impacts of tax in Seattle, by income level

#### Question-specific results

Lower-income (52%; 95% CI: 46, 58%), versus higher-income (63%; 95% CI: 58, 68%), respondents were less supportive of the tax, although this difference was not statistically significant after correction for multiple comparisons (Table 2). Lower-income respondents were also more concerned about the potential negative consequences of the tax, particularly in regard to whether the tax would improve public health, result in job loss, affect family finances and result in cross-border shopping. There was a statistically significant difference in perceptions of how the tax would affect public health. Fewer lower-income participants (48%; 95% CI: 42, 53%), compared to higher-income (61%; 95% CI: 55, 66%), perceived that the tax would improve public health. There was also a statistically significant difference in perceptions of how the tax would affect job loss and family finances. Fewer lower-income participants (58%; 95% CI: 53, 64%), compared to higher-income (71%; 95% CI: 66, 75%), perceived that the tax would not result in job loss. Similarly, 68% (95% CI: 62, 73%) of lower-income participants believed that the tax would not negatively affect their family’s finances, which was lower than higher-income participants (85%; 95% CI: 81, 88%). We also observed significant differences in perceptions between lower- and higher-income individuals regarding whether or not they would leave Seattle to purchase sugary beverages to avoid the tax. This difference was driven by the fact that a higher proportion of lower-income participants (6.1%; 95% CI: 3.7, 9.9%), compared to higher-income (1.0%; 95% CI: 0.39, 2.4%), responded that they “don’t know” in response to our question about cross-border shopping. However, a qualitatively lower proportion of lower-income participants (72%; 95% CI: 67, 77%), versus higher-income (80%; 95% CI: 76, 84%), also reported that they would not leave Seattle to purchase sugary beverages to avoid the tax.

#### Overall tax impact score

Descriptive statistics for the overall tax impact score are presented in Table 3. In unadjusted models, lower-income participants, compared to higher-income, had a significantly lower tax impact score, which is interpreted as having a less positive (or more negative) perception of the sugary beverage tax (β = − 1.4; 95% CI: - 2.2, − 0.67) (Table 3). Results were similar in multivariate models, but they did not reach statistical significance when adjusting for multiple comparisons (β = − 0.98; 95% CI: − 1.8, − 0.19).

**Table 3:** Perceived Positive Impacts of the Tax, by Income Level and Race/Ethnicity

	Perceived Positive Impacts of the Sugary Beverage Tax	Unadjusted Perceived Positive Impacts of Sugary Beverage Tax^1^		Adjusted Perceived Positive Impacts of Sugary Beverage Tax^1,2^	
	Mean Score (CI)	β (95% CI)	*p* value	β (95% CI)	*p* value
Income Level					
<260% FPL	1.9 (1.3, 2.4)	−1.4 (−2.2, −0.67)	<0.001*	− 0.98 (− 1.8, − 0.19)	0.015
≥260% FPL	3.3 (2.8, 3.8)	reference		reference	
Race/Ethnicity					
Non-Hispanic White	3.0 (2.5, 3.5)	reference	0.49^4^	reference	0.85^4^
Non-Hispanic Black/African American	2.1 (0.93, 3.3)	−0.84 (−2.1, 0.46)		−0.13 (−1.5, 1.2)	
Non-Hispanic Asian	2.5 (1.2, 3.8)	−0.51 (−1.8, 0.86)		−0.50 (− 1.8, 0.84)	
Non-Hispanic Other^3^	2.2 (1.0, 3.3)	−0.83 (−2.0, 0.40)		−0.63 (−1.9, 0.65)	
Hispanic	2.3 (0.70, 3.8)	−0.71 (−2.3, 0.93)		0.10 (−1.5, 1.7)	
*Constant*		3.0 (2.5, 3.5)		4.4 (2.3, 6.5)	

*CI* confidence interval

^1^Estimated using linear regression models with robust standard errors. Compared to the reference population, a lower score can be interpreted as a less positive perception of tax impacts

^2^In addition to mutually adjusting for income and race/ethnicity, models control for education, sex, age, political affiliation

^3^People who are Native Hawaiian or Other Pacific Islander, American Indian and Alaska Natives, or two or more races are categorized as non-Hispanic Other

^4^Global *p* values that compare all race/ethnicities to each other

* Perceived positive impacts of sugary beverages among lower-income participants is statistically significantly different from perceived positive impacts of sugary beverages among higher-income participants, after considering multiple comparisons, using the Holm-Bonferroni Sequential Correction (*p* < 0.01)

### Perceived impacts of tax in Seattle by race/ethnicity

#### Question-specific results

Overall, people of color were qualitatively less supportive of the tax, although many of these differences in point estimates were not statistically significant (Table 4). In addition, people of color (compared to non-Hispanic whites) were qualitatively less confident that the tax would improve health outcomes and were somewhat more concerned about the potential economic downsides of the tax. But, only the perception that the tax may impact one’s own family finances reached statistical significance for differences by race/ethnicity (global *p* < 0.001); compared to non-Hispanic Whites (82%; 95% CI: 79, 86%), a smaller proportion of non-Hispanic Blacks (63%; 95% CI: 48, 75%), and Hispanics (67%; 95% CI: 51, 79%), perceived that the tax would not negatively affect their family finances.

**Table 4:** Perceived Health and Economic Impacts of Tax in Seattle, by Race/Ethnicity

	Non-Hispanic Whites^a^ (*N* = 588)	Non-Hispanic Blacks^a^ (*N* = 60)	Non-Hispanic Asians^a^ (*N* = 66)	Non-Hispanic Other Race^a,b^ (*N* = 78)	Hispanics (*N* = 56)^a^	*p* value
	% (95% CI)	% (95% CI)	% (95% CI)	% (95% CI)	% (95% CI)	
Opinion on Tax^c^						
Approve	64% (59, 68%)	46% (33, 60%)	49% (36, 62%)	53% (41, 65%)	53% (38, 68%)	0.17
Disapprove	33% (28, 37%)	49% (36, 63%)	45% (32, 58%)	42% (31, 55%)	42% (28, 57%)	
Don’t know	3.9% (2.5, 6.1%)	4.3% (1.0, 17%)	6.4% (2.4, 16%)	4.4% (1.6, 12%)	5.1% (1.3, 18%)	
Child Well Being^d^						
Tax will improve child health and wellbeing	61% (57, 66%)	46% (33, 61%)	59% (46, 71%)	44% (33, 57%)	60% (45, 73%)	0.18
Tax will not improve child health and wellbeing	35% (31, 40%)	44% (31, 58%)	36% (25, 50%)	51% (38, 63%)	39% (26, 54%)	
Don’t know	3.3% (2.0, 5.3%)	10% (3.6, 23%)	4.5% (1.5, 13%)	4.9% (1.4, 16%)	1.2% (0.17, 8.2%)	
Public Health^d^						
Tax will improve public health	59% (54, 63%)	42% (29, 57%)	55% (42, 68%)	42% (30, 54%)	52% (38, 67%)	0.095
Tax will not improve public health	38% (33, 42%)	48% (34, 62%)	39% (27, 52%)	51% (39, 64%)	48% (33, 62%)	
Don’t know	3.6% (2.3, 5.6%)	10% (3.7, 24%)	5.6% (2.1, 14%)	6.8% (2.6, 17%)	0% (0, 0%)	
Cross-Border Shopping^d^						
Participant will not cross-border shop	78% (74, 82%)	77% (63, 87%)	77% (64, 86%)	82% (70, 90%)	68% (52, 80%)	0.029
Participant will cross-border shop	20% (17, 25%)	13% (7.0, 25%)	17% (10, 29%)	17% (10, 29%)	29% (17, 45%)	
Don’t know	1.7% (0.91, 3.0%)	10% (3.6, 23%)	5.8% (2.2, 15%)	1.0% (0.10, 6.7%)	3.3% (0.80, 13%)	
Seattle's Economy^d^						
Tax will have a positive effect on the economy	48% (43, 53%)	49% (35, 63%)	46% (33, 59%)	44% (32, 56%)	50% (35, 64%)	0.81
Tax will have a negative effect on the economy	36% (31, 40%)	26% (16, 39%)	39% (27, 53%)	34% (23, 47%)	35% (23, 51%)	
Don’t know	16% (13, 20%)	25% (14, 39%)	15% (7.8, 27%)	22% (13, 34%)	15% (7.7, 27%)	
Small Businesses^d^						
Tax will not negatively affect small businesses	53% (49, 58%)	45% (32, 59%)	51% (38, 64%)	42% (31, 55%)	60% (44, 73%)	0.036
Tax will negatively affect small businesses	40% (35, 44%)	38% (25, 52%)	33% (22, 46%)	48% (36, 60%)	38% (25, 53%)	
Don’t know	7.1% (5.3, 9.5%)	17% (8.6, 31%)	17% (9.1, 29%)	10% (4.3, 21%)	2.4% (0.59, 9.3%)	
Job Loss^d^						
Tax will not result in job loss	67% (62, 71%)	61% (46, 73%)	63% (50, 75%)	65% (52, 75%)	75% (60, 86%)	0.022
Tax will result in job loss	25% (21, 29%)	22% (13, 34%)	16% (9.0, 29%)	19% (11, 31%)	19% (10, 34%)	
Don’t know	8.2% (6.2, 11%)	18% (9.1, 31%)	20% (12, 33%)	16% (9.0, 27%)	5.3% (1.6, 16%)	
Family Finances^d^						
Tax will not negatively affect family finances	82% (79, 86%)	63% (48, 75%)	73% (60, 83%)	81% (71, 88%)	67% (51, 79%)	<0.001*
Tax will negatively affect family finances	16% (13, 20%)	31% (20, 45%)	16% (8.0, 27%)	14% (8.0, 24%)	27% (15, 43%)	
Don’t know	1.3% (0.70, 2.4%)	6.4% (1.9, 20%)	11% (5.3, 22%)	4.6% (1.7,12%)	5.6% (1.8, 17%)	
Impact on Low-Income People and People of Color						
Tax will positively impact people with low-income/people of color	50% (45, 54%)	54% (40, 68%)	37% (25, 50%)	46% (34, 59%)	49% (34, 64%)	0.48
Tax will negatively impact people with low-income/people of color	40% (36, 45%)	32% (20, 46%)	48% (35, 61%)	42% (31, 55%)	44% (30, 59%)	
Don’t know	10% (7.8, 13%)	14% (6.6, 26%)	15% (7.9, 27%)	11% (5.3, 22%)	7.3% (2.7, 19%)	
Individual Choice^d^						
People will have the choice to drink the beverages they want	74% (70, 78%)	64% (49, 76%)	63% (50, 75%)	71% (59, 81%)	63% (48%,7 6%)	0.32
People will not have the choice to drink the beverages they want	23% (20, 27%)	33% (22, 47%)	32% (21, 46%)	24% (15, 36%)	37% (24, 52%)	
Don’t know	2.8 (1.7, 4.4%)	3.0% (0.42, 18%)	4.5% (1.5, 13%)	4.7% (1.3, 15%)	0% (0.0, 0.0%)	

*CI* confidence interval

^a^Values are rounded to two significant digits. Missing data: ethnicity (*n* = 3 [individuals who responded “don’t know”]); opinion on tax (*n* = 1); cross-border shopping (*n* = 4), small business (*n* = 1); job loss (*n* = 1); impact on people with low-income/people of color (*n* = 4); individual choice (*n* = 1)

^b^Native Hawaiian or Other Pacific Islanders, American Indian and Alaska Natives, and those reporting two or more races are categorized as non-Hispanic Other

^c^Responses included: strongly disapprove, somewhat disapprove, somewhat approve, strongly approve, don’t know. These categories are collapsed into: approve, disapprove, don’t know

^d^Participants were read two statements and asked to indicate if the first statement was much closer, the first statement was somewhat closer, the second statement was much closer, or the second statement was somewhat closer. There was also an option to report “don’t know” or “refused” for each of the questions. These categories are collapsed into: the first statement was closer, the second statement was closer or don’t know

^*^Denotes statistically significant difference in participants’ response to all three response categories (agree, disagree, don’t know), comparing all races to each other, after considering multiple comparisons, using the Holm-Bonferroni Sequential Correction (global *p* < 0.01). Statistical significance estimated using a Chi-squared test

#### Overall tax impact score

There were not statistically significant differences in the tax impact summary score by race/ethnicity in unadjusted (non-Hispanic Black β = − 0.84, 95% CI: − 2.1, 0.46; non-Hispanic Asian β = − 0.51, 95% CI: − 1.8, 0.86; non-Hispanic Other β = − 0.83, 95% CI: − 2.0, 0.40, Hispanic β = − 0.71, 95% CI: − 2.3, 0.93) or adjusted models (non-Hispanic Black β = − 0.13, 95% CI: − 1.5, 1.2; non-Hispanic Asian β = − 0.50, 95% CI: − 1.8, 0.84; non-Hispanic Other β = − 0.63, 95% CI: − 1.9, 0.65), Hispanic β = 0.10, 95% CI: − 1.5, 1.7) (Table 3).

#### Supplemental analysis

Approximately 65% of females supported the tax, compared to 54% of males (Additional file 1: Table S4). Support for the tax was similar across age groups, but participants with a higher level of education tended to have higher levels of support for the tax, as did those who self-reported that they were Democrats (67%), compared to Independents (51%) or Republicans (47%). Support was similar irrespective of whether participants had knowledge of the tax prior to completing the survey. About 70% of participants who never or rarely consumed sugary beverages (none or < 1 per-week) supported the tax, compared to only 52% of participants who consumed sugary beverages at least one time per-week.

## Discussion

This study aimed to better understand the public’s perceptions of the possible impacts of the tax on health and economics in Seattle, as concerns about potential unintended impacts, particularly on people of color and people with low-income, emerged during the public debate on Seattle’s tax. We find that a majority of participants support the sugary beverage tax in Seattle and correspondingly, most people believed that the tax will positively impact health, and will not negatively affect general and personal economics in Seattle. However, lower-income, versus higher-income, respondents were more concerned about the possible negative economic consequences of the tax (e.g. job loss, personal finances). Although we observed that people of color had a qualitatively lower level of support, only the perception that the tax may have unintended consequences for one’s own family finances significantly differed by race/ethnicity.

In Seattle, 59% of participants supported the tax. We are cautious to compare our results to other polls because public support for sugary beverage taxes throughout the United States has varied depending on question wording, geographic location, the revenue target for the tax (e.g., general fund versus programs addressing healthy eating, etc), and the year conducted. In this survey, we prefaced our question regarding support for the tax by explaining what the tax revenue would be used for in Seattle: access to healthy and affordable food and expanding access to education (see Additional file 3). Several prior surveys also asked individuals about support, while concurrently explaining how the revenue would be used, usually to improve health programs and services. In these surveys, support in Seattle was similar to that reported in California (62%) [24], Vermont (60%) [25], and higher than support in one mid-Atlantic state (50%) [13]. Notably, prior studies suggest that sugary beverage consumption [7] and political affiliation [12, 26] are related to support for sugary beverage taxes. In Seattle, consumption of at least one sugary beverage per-day (17%) was lower than prior estimates, which included 23 states and suggested that 31% of U.S. adults consume one or more sugary beverage per-day [21] and a majority of participants reported that they are Democrats (as are a majority of Seattle residents), who tend to be more supportive of sugary beverage taxes [12, 26]. Lower levels of consumption and the political affiliation of many participants in Seattle could be related to the overall high levels of support for the tax.

Similar to the overall level of support for the tax, we find that a majority (56%) of participants perceived that the tax would improve public health. We do not test whether individual-level support was related to perceived health impacts; but, our findings are generally consistent with prior literature that suggests support for sugary beverage taxes is aligned with perceived health impacts of the tax and more generally, perceived healthfulness of sugary beverages. In one mid-Atlantic state, believing that sugary beverages contribute to obesity (versus not) was associated with 3-fold higher odds of supporting a sugary beverage tax [13]. In a nationally representative sample of France, Julia and colleagues find that 73% of respondents supported a sugary beverage tax when they were told that the revenue would be used to improve the health-care system, compared to only 50% when the use of the tax revenue was not made explicit [7]. Consistent with these surveys that poll constituents, key stakeholders (e.g. advocates, policymakers) in California perceived that the most effective messages were those that emphasized reinvesting tax revenue into health-related programs and linking sugary beverage consumption to obesity and diabetes [27]. Relatedly, believing that obesity is driven by environmental factors, like the availability and price of sugary beverages, has been associated with higher levels of support for a sugary beverage tax [8, 14].

Respondents were less certain about their perceptions regarding how the tax would impact the Seattle economy broadly, as indicated by the higher proportion people responding “don’t know” to that question (~ 20%). But, in general, we found perceptions about economic effects to be consistent with tax support. Although common arguments against sugary beverage taxes include that they will negatively impact the economy [8], a majority of participants believed that the tax would not negatively affect small businesses (52%), employment (66%), or their own finances (79%). Two prior studies find that sugary beverage taxes are not associated with job loss. Research from Mexico suggests that there were not significant changes in employment in the manufacturing industries or commercial stores after the implementation of a sugary beverage tax [28]. Using a macroeconomic simulation model to assess the employment impact of a 20% sugary beverage tax, Powell and colleagues found increased employment in Illinois and California overall after a sugary beverage tax was implemented; some declines in employment within the beverage industry were offset by new employment in non-beverage industry and government sectors [29].

Our findings suggest that there are lower levels of support for sugary beverage taxes among lower-income (versus higher-income) populations. Although differences in support for the tax by income level were not statistically significant when adjusting for multiple comparisons, 63% of high-income respondents approved of the tax, compared to only 52% of lower-income participants. Some prior studies also suggest socioeconomic status is related to support for sugary beverage taxes [12–14]. But these prior results are mixed. For example, contrary to our results, Gollust and colleagues [12] ﻿report that those with higher incomes expressed lower support for sugary beverage taxes, whereas Donaldson and colleagues report that having a graduate level of education, compared to high school level, is associated with a 2-fold higher odds of supporting a sugary beverage tax [13]. Notably, neither of these surveys were fielded in locations where a tax had been (or was going to be) implemented, so individuals were not exposed to tax messaging campaign, whereas in Seattle, there was a focus on regressivity in media during the year prior to tax implementation. In our sample, aligned with lower levels of tax support, lower-income participants, compared to higher-income, perceived significantly less positive (or more negative) impacts of the sugary beverage tax in Seattle. Lower proportions of lower-income participants perceived that the tax would not result in job loss nor would it negatively affect their family’s finances and only 48% of lower-income participants perceived that the tax would improve the public’s health, compared to 61% of higher-income participants. Julia and colleagues also report that lower education respondents (versus higher education), had lower odds of perceiving that the sugary beverage tax in France would improve public health [7]. Julia and colleagues also find that a larger proportion of lower-income participants (versus higher-income), perceived that a sugary beverage tax in France would “unfairly” impact lower-income populations [7]. We did not find that perceptions differed by income level on whether the tax would disproportionately impact low-income people and/or people of color. Moreover, studies that have assessed the extent to which a sugary beverage tax could disproportionately affect low-income populations find that the burden on lower- and higher-income populations is virtually the same [30]. Models that use simulations to estimate the effects of sugary beverage taxes show that lower-income households would pay 0.10 to 1.0% of their annual income in a sugary beverage tax, compared to 0.03 to 0.60% of annual income among higher income households, equating to a difference of ﻿approximately $5 per year [30].

Based on prior studies that find sugary beverage consumption varies by race/ethnicity [20, 21, 31, 32] and that consumption is related to support for sugary beverage taxes [7], we hypothesized that perceptions of the tax and tax impacts would differ by race/ethnicity. People of color were qualitatively less supportive of the tax, less confident that the tax would improve health outcomes, and somewhat more concerned about the potential economic downsides of the tax. But differences in the distribution of responses by race/ethnicity largely did not reach statistical significance. This is consistent with two prior studies, one random-digit dialed telephone sample of U.S. adults, conducted in 2009–2010 [9], and one representative household survey of Kansas residents in 2014 [14], that report similar levels of support for sugary beverage taxes when comparing Black or Hispanic populations to White populations. But it is plausible that the present study was underpowered to detect differences by race/ethnicity, as our primary objective was to be able to investigate differences by income level.

A majority of people supported the tax in Seattle and the general perception was that the impacts of the tax will be positive. Although large proportions of respondents with lower-income and/or people of color also supported the tax, some Seattle residents were concerned about possible unintended consequences of the tax on job loss, the economy, and small businesses. A proportion of the public was also unsure if the tax will benefit public health and child health and wellbeing. These concerns were more common among people with lower-income.

There are several implications of our findings for practice. Some concerns about regressive tax impacts, as well as negative economic consequences (e.g. family finances), suggests that sugary beverage tax policies should be designed to mitigate these issues through the dedication of revenues to address health and social inequities. It is also important to address concerns about unintended consequences by developing clear messaging to inform constituents and business owners about the policy goals of the tax, engage the community in decisions about how to invest tax revenues, and report on use the of revenues and benefits from funded activities. Monitoring impacts on jobs and small business revenues will also be important.

These findings should be interpreted while keeping in mind the limitations of our study. First, the final response rate to our survey was about 5%. However, this is similar to a recent survey conducted in Philadelphia that queried respondents on sugary beverage taxes [5] and even with low response rates, phone surveys that include landline and cell phones, and that are adjusted to match demographic profile of Seattle can accurately estimate perceptions [15, 16]. Sample weights were utilized in all analyses, based on the race/ethnicity, gender, median household income and the age distribution of Seattle. However, sample weights did not take into account non-response or different response rates for telephone versus online respondents. Like many surveys and opinion polls, these data are subject to selection bias, in that those who choose to participate are likely different from those who do not participate. We also cannot assess whether respondents and non-responders differed substantially in terms of their demographic characteristics or in their support for sugary beverage taxes. If responding to the survey is correlated with opinions about the sugary beverage tax, it could bias the study findings. Although we do provide the respondents with information regarding how the tax will affect the price per ounce, it is plausible that respondents were not aware of how much the tax would increase the total price of a sugary beverage. A better understanding of the total price (or the price increase) could affect individuals’ support for the tax and perceptions about tax impacts. These data are cross-sectional, therefore, we cannot infer that any associations are causal and there may be unmeasured confounders. Generalization of our results to other cities may be limited. Finally, our survey was not validated and the results may be specific to the use of this particular scale.

Despite these limitations, we examine support for Seattle’s sugary beverage tax in a relatively large sample of adults and to our knowledge, this is the first study to survey adults on their perceptions of the possible economic impacts of sugary beverage taxes. We also provide important insights into the perceptions of the tax and tax impacts by income level and race/ethnicity.

## Conclusions

The sustainability of sugary beverage taxes may depend on the public’s support of the tax itself and the public’s perception of the possible impacts of the tax. We find that a majority of participants support the sugary beverage tax in Seattle and correspondingly, most people perceived that the tax would positively impact health, and would not have negative economic effects. People with lower-income, versus higher-income, were somewhat more concerned about the possible negative economic consequences of the tax, although still largely supportive of the tax. Future studies should assess whether heightened media attention during the course of adopting and implementing a sugary beverage tax, as well as people’s experience with the tax once implemented, changes the public’s perceptions over time. Further evaluation of the extent to which unintended consequences (e.g. job loss) occur is also needed.

## Additional files

Additional file 1: Includes 4 supplemental tables that detail 1) Response rate for phone interviews, 2) Mode of completion by income and race/ethnicity and 3) Demographic characteristics of survey participants in Seattle compared to American Community Survey for the City of Seattle, and 4) Perceived support for the tax in Seattle by selected characteristics. (DOCX 20 kb)

Additional file 2: Provides additional information on the raking method. (DOCX 12 kb)

Additional file 3: Includes the modified survey instrument. (DOCX 38 kb)

## Data Availability

The de-identified dataset used during the current study is available from the corresponding author upon reasonable request.
