# Supplementary material for: Perceptions of the possible health and economic impacts of Seattle’s sugary beverage tax
Source: BMC Public Health. 2019 Jul 9;19:910. doi: 10.1186/s12889-019-7133-2 (PMC6617661; doi:10.1186/s12889-019-7133-2)
Supplement: Supplementary file 1 — Includes 4 supplemental tables that detail 1) Response rate for phone interviews, 2) Mode of completion by income and race/ethnicity and 3) Demographic characteristics of survey participants in Seattle compared to American Community Survey for the City of Seattle, and 4) Perceived support for the tax in Seattle by selected characteristics. (DOCX 20 kb) [file 12889_2019_7133_MOESM1_ESM.docx]

**Additional File 1**

| **Supplemental Table 1. Response Rate for Phone Interviews^1^** | | |
| --- | --- | --- |
|  | Landline | Cell |
| Total Sample Available | 11,312 | 18,696 |
| Completed Surveys | 93 | 328 |
| Unable to Reach | 8,082 | 10,992 |
| Refused to Participate | 1,685 | 3,512 |
| Partial Completion | 6 | 50 |
| Excluded due to zipcode or age | 170 | 132 |
| Excluded due to screening qualifications^2^ | 23 | 117 |
| Bad Numbers | 1,253 | 3,565 |
| AAPOR Response Rate 4^3^ | 3.6% | 6.7% |

^1^ A response rate was not available for the online version of the survey.

^2^ Respondents were excluded if they refused to answer screening questions on income and race/ethnicity.

^3^ AAPOR Response Rate 4 = (complete interviews + partial interviews) / (complete interviews + partial interviews + refusals + non-contact + other non-interview + [the % of known-residential cases estimated to have eligible respondent * the % of unknown-if-residential cases that are estimated to be residential * unknown residential] + [the % of unknown-if-residential cases that are estimated to be residential * unknown]).

| **Supplemental Table 2. Mode of completion by income and race/ethnicity** | | |
| --- | --- | --- |
|  | **Phone (N=421)** | **Web (N=430)** |
|  | N (weighted %) | N (weighted %) |
| **Income Level** |  |  |
| < 260 % FPL | 177 (35%) | 218 (65%) |
| ≥ 260% FPL | 244 (48%) | 212 (52%) |
| **Race/Ethnicity^2^** |  |  |
| Non-Hispanic White | 295 (45%) | 293 (55%) |
| Non-Hispanic Black/African American | 33 (50%) | 27 (50%) |
| Non-Hispanic Asian | 20 (30%) | 46 (70%) |
| Non-Hispanic Other^2^ | 44 (54%) | 34 (46%) |
| Hispanic | 29 (44%) | 27 (56%) |

**^1^** N is unweighted to show actual sample size whereas percentages (%) are based on weighted to the ACS (2012-2016). Therefore, the percentages displayed will be different from the number you get by dividing the total N by the cell-specific N.

^2^ People who are Native Hawaiian or Other Pacific Islander, American Indian and Alaska Natives, or two or more races are categorized as non-Hispanic Other.

**Supplemental Table 3. Demographic characteristics of survey participants in Seattle compared to American Community Survey for City of Seattle**

|  | **N** | **Sample**  **Unweighted %** | **Sample Weighted %** | **ACS**  **(2012-2016) %^1^** |
| --- | --- | --- | --- | --- |
| **Gender** |  |  |  |  |
| Male | 349 | 41% | 50% | 50% |
| Female | 499 | 59% | 50% | 50% |
| **Race/Ethnicity** |  |  |  |  |
| Non-Hispanic White | 588 | 69% | 66% | 66% |
| Non-Hispanic Black/African American | 60 | 7.1% | 7.0% | 7.0% |
| Non-Hispanic Asian | 66 | 7.8% | 14% | 14% |
| Non-Hispanic Other**^2^** | 78 | 9.2% | 6.7% | 6.7% |
| Hispanic | 56 | 6.6% | 6.6% | 6.6% |
| **Age** |  |  |  |  |
| 18-64 years old | 588 | 70% | 86% | 86% |
| ≥ 65 years old | 250 | 30% | 14% | 14% |
| **Household Level Income (annual)** |  |  |  |  |
| <$30,000 | 242 | 30% | 21% | 21% |
| $30,000-59,999 | 213 | 26% | 21% | 21% |
| ≥ 60,000 | 355 | 44% | 58% | 58% |

ACS = American Community Survey

^1^ Categories for race/ethnicity, age, and household level income are collapsed to facilitate comparisons between our sample and the ACS.

^2^ People who are Native Hawaiian or Other Pacific Islander, American Indian and Alaska Natives, or two or more races are categorized as non-Hispanic Other.

**Supplemental Table 4. Perceived Support of Tax in Seattle by Selected Characteristics^1^**

|  | Approve | Disapprove | Don’t Know |
| --- | --- | --- | --- |
|  | % (95% CI) | % (95% CI) | % (95% CI) |
| **Gender** |  |  |  |
| Male (N=349) | 54%  (48%, 59%) | 42%  (36%, 48%) | 4.6%  (2.6%, 8.0%) |
| Female (N=498) | 64%  (59%, 69%) | 32%  (27%, 37%) | 4.1%  (2.6%, 6.6%) |
| **Age** |  |  |  |
| 18-30 years (N=133) | 62%  (52%, 71%) | 35%  (26%, 44%) | 3.5%  (1.4%, 8.9%) |
| 31-40 years (N=152) | 59%  (51%, 67%) | 36%  (28%, 44%) | 4.8%  (2.2%, 10%) |
| 41-50 years (N=136) | 58%  (49%, 67%) | 36%  (27%, 45%) | 6.0%  (2.8%, 12%) |
| 51-64 years (N=167) | 57%  (49%, 65%) | 40%  (32%, 48%) | 3.1%  (1.3%, 7.5%) |
| ≥ 65 years (N=249) | 58%  (51%, 65%) | 37%  (31%, 44%) | 4.6%  (2.4%, 8.7%) |
| **Education Level** |  |  |  |
| Some high school (N=24) | 43%  (24%, 64%) | 50%  (24%, 70%) | 7.6%  (1.9%, 26%) |
| Completed high school (N=79) | 42%  (30%, 55%) | 54%  (42%, 66%) | 3.5%  (1.0%, 14%) |
| Some college/vocational training (N=198) | 49%  (41%, 57%) | 47%  (39%, 55%) | 4.9%  (2.4%, 9.9%) |
| Completed college or university (N=294) | 63%  (57%, 69%) | 32%  (26%, 38%) | 4.8%  (2.7%, 8.5%) |
| Completed graduate degree (N=241) | 66%  (59%, 73%) | 31%  (24%, 38%) | 3.5%  (1.6%, 7.3%) |
| **Prior Knowledge of Tax** |  |  |  |
| Participant had *not* heard of the tax (N=198) | 60%  (52%, 68%) | 35%  (28%, 43%) | 4.8%  (2.4%, 9.4%) |
| Participant had heard of the tax (N=634) | 59%  (54%, 63%) | 38%  (34%, 43%) | 3.0%  (1.8%, 5.0%) |
| **Political Affiliation** |  |  |  |
| Democrat (N=462) | 67%  (62%, 72%) | 29%  (24%, 34%) | 3.7%  (2.2%, 6.2%) |
| Independent (N=236) | 51%  (44%, 58%) | 46%  (39%, 54%) | 2.4%  (1.1%, 5.4%) |
| Republican (N=71) | 47%  (34%, 60%) | 47%  (34%, 60%) | 6.0%  (2.0%, 17%) |
| **Sugary Beverage Consumption** |  |  |  |
| None or < 1 per week (N=405) | 68%  (62%, 73%) | 27%  (22%, 32%) | 5.3%  (3.2%, 8.6%) |
| ≥ 1 per week (N=445) | 52%  (46%, 57%) | 45%  (39%, 50%) | 3.7%  (2.2%, 6.2%) |

CI = confidence interval

^1^ Values are rounded to two significant digits. Missing data: gender (n=4), age (n=14), education (n=15), prior knowledge of tax (n=1); political affiliation (n=1), consumption (n=1).
