# Supplementary material for: Perceptions of the possible health and economic impacts of Seattle’s sugary beverage tax
Source: BMC Public Health. 2019 Jul 9;19:910. doi: 10.1186/s12889-019-7133-2 (PMC6617661; doi:10.1186/s12889-019-7133-2)
Supplement: Supplementary file 2 — Provides additional information on the raking method. (DOCX 12 kb) [file 12889_2019_7133_MOESM2_ESM.docx]

**Additional File 2**

**Raking Method**

The raking method is an iterative, proportional weighting method. The researcher selects a set of variables for which 1) they would like to weight the data and 2) the population distribution for those variables is known (e.g. sex, race/ethnicity, income). For example, one could specify that the sample should be 65.7% non-Hispanic White, 7.0% non-Hispanic Black, 14.1% non-Hispanic Asian, and 6.7% non-Hispanic Other, because it is being drawn from a geographic area in which this is the racial/ethnic distribution of the population. Then, using the Stata command *ipfweight*, the procedure iteratively adjusts the weight for each study respondent until the sample distribution aligns with the population distribution for that variable specified (e.g. race/ethnicity). The procedure is subsequently performed for each of the additional variables that the researcher has selected (e.g. sex, income, and so on). If the second adjustment, for sex, alters the race/ethnicity distribution such that it is no longer the same as the population distribution, then the weights are adjusted again. The process is repeated until the weighted distribution of all of the weighting variables matches their specified targets.
