# Supplementary material for: Perceptions of the possible health and economic impacts of Seattle’s sugary beverage tax
Source: BMC Public Health. 2019 Jul 9;19:910. doi: 10.1186/s12889-019-7133-2 (PMC6617661; doi:10.1186/s12889-019-7133-2)
Supplement: Supplementary file 3 — Includes the modified survey instrument. (DOCX 38 kb) [file 12889_2019_7133_MOESM3_ESM.docx]

# **Additional File 3**

# **Modified Survey Questionnaire**

# **Phone Version^[[1]](#footnote-1)^**

Hello, my name is ___________. I’m working with the University of Washington and I am looking for someone to answer some questions about the sugary drink tax that will start in January in Seattle. There are no right or wrong answers and your answers will be kept confidential. Do you have a few minutes to answer some brief questions?

INTERVIEWER NOTE: *if needed, the survey will take about 15 minutes to complete.*

# **Screener Questions**

First, I’d like to ask you a few questions about your household to make sure you are eligible for this survey.

1. Can you tell me what zip code you live in? _______________________

**INTERVIEWER NOTES:**

**IF respondent does not live in any of the zip codes listed below, TERMINATE**

**IF respondent lives in a zip code entirely within city limits CONTINUE**

**If respondent lives in zip code that borders Northern city limits ask question 2**

**If respondent lives in zip code that borders Southern city limits as question 3**

**If DK OR REFUSED - TERMINATE**

Zip codes clearly in Seattle city limits: 98101, 98102, 98103, 98104, 98105, 98107, 98109, 98112, 98115, 98116, 98119, 98121, 98122, 98125, 98126, 98134, 98144, 98154, 98164, 98174, 98177, 98195, 98199

Zip codes the overlap Seattle city limits in North: 98133, 98117

Zip codes the overlap Seattle city limits in South: 98146, 98136, 98106, 98108, 98118, 98178

1. Do you live above or below 145^th^ street?

🞎 Above [TERMINATE]

🞎 Below [CONTINUE]

DK/REFUSED - TERMINATE

1. Do you live within Seattle city limits?

🞎 No [TERMINATE]

🞎 Yes [CONTINUE]

DK / REFUSED - TERMINATE

1. Are you of Hispanic or Latino origin? (Check all that apply)

🞎 No, not of Hispanic, Latino or Spanish origin

🞎 Yes, Mexican, Mexican American or Chicano

🞎 Yes, Puerto Rican

🞎 Yes, Cuban

🞎 Yes, another Hispanic, Latino or Spanish origin

🞎 DON’T KNOW

🞎 REFUSED

1. What race(s) do you consider yourself? (Check all that apply)

🞎 White

🞎 Black or African American

🞎 American Indian or Alaska Native (ASSIGN TO OTHER)

🞎 Asian

🞎 Native Hawaiian or Other Pacific Islander (ASSIGN TO OTHER)

🞎 Other _________________________

🞎 DON’T KNOW - TERMINATE

🞎 REFUSED - TERMINATE

1. How many adults (including yourself) live in your household? _______ adults

IF DK/REFUSED - TERMINATE

1. How many children under 18 live in your household? ________ children

IF DK/REFUSED TERMINATE

1. Is your total annual household income above or below_________ per year?

IF DK/REFUSED TERMINATE

INTERVIEWER NOTE: use chart to get household size specific value for 260% FPL for this household

🞎 Above (“high” income)

🞎 Below (“low” income)

| **PROGRAMMING INSTRUCTIONS**  **Household Size**  **Add Q2+Q3** | **Annual 260%**  **Insert in Q8** |
| --- | --- |
| 1 | $ 31,356 |
| 2 | $ 42,224 |
| 3 | $ 53,092 |
| 4 | $ 63,960 |
| 5 | $ 74,828 |
| 6 | $ 85,696 |
| 7 | $ 96,564 |
| 8 | $ 107,432 |

INTERVIEWER: READ DRINK TYPES IN BOLD ONLY – READ BRANDS IN PARENTHESIS ONLY IF NEEDED

Because we will be talking today about sugary drinks, I want to start off by telling you what we mean when we refer to sugary drinks. **Sugary drinks** include **regular soft drinks, soda or pop** (such as Coke, Pepsi, Sprite, Root Beer, Orange Soda, Jarritos, Dr. Pepper), **fruit-flavored drinks** (such lemonade, Sunny Delight, Hawaiian Punch), **sports drinks** (such as Gatorade, Powerade), **sweetened teas or coffees** (such as Arizona Iced Tea, Snapple, Pure Leaf, Starbucks Frappuccino, mochas, or bubble teas), and **energy drinks** (such as Red Bull, Rockstar, Monster). They do NOT include milk, 100% fruit juice, diet drinks, or artificially sweetened drinks.

To start off, I’m interested in learning about whether you drink sugary drinks.

- 1. During the past 30 days, did you drink **sugary drinks** never or less than 1 time per week, 1 time per week, 2-6 times per week, 1 time per day, or 2 or more times per day?

🞎 Never or less than 1 time per week

🞎 1 time per week

🞎 2-6 times per week

🞎 1 time per day

🞎 2 or more times per day

🞎 Don't know

🞎 REFUSED

# **Domain 1: Norms/Attitudes towards tax itself**

Next, I’d like to tell you a little bit about the new tax on sugary drinks in Seattle.

Starting on January 1, 2018, the City of Seattle will start taxing sugary drinks. In Seattle, large distributors will now pay a 1.75 cents per ounce tax on sugary drinks. Taxed beverages include drinks that have added sugar. The tax will NOT include diet beverages, 100% fruit juices, or milk products. Money from the tax will help give more people access to healthy and affordable food, expand early education for pre-school aged kids, and help high school graduates enter college.

- 1. Have you heard of this tax, yes or no?

🞎 Yes

🞎 No

🞎 Don’t know

🞎 REFUSED

- 1. Based on what you know, do you strongly disapprove, somewhat disapprove, somewhat approve, strongly approve of this tax?

🞎 Strongly disapprove

🞎 Somewhat disapprove

🞎 Somewhat approve

🞎 Strongly approve

🞎 Don’t know

🞎 REFUSED

- 1. I’m going to read you pairs of statements that people have made about this new tax on sugary drinks. After I read each pair, please tell me which statement is closer to your own view, even if neither is exactly right.

**(INTERVIEWER PROMPT)** Which statement comes closer to your own view?

| ____ 4A | 1. This tax WILL improve public health in Seattle.  2. This tax will NOT improve public health in Seattle. |
| --- | --- |
| ____ 4B | 1. This tax WILL improve the health and well-being of children in Seattle.  2. This tax will NOT improve the health and well-being of children in Seattle. |

**(AFTER CHOICE IS MADE, INTERVIEWER PROBE:)** Is that MUCH closer or SOMEWHAT closer?

🞎 FIRST statement is MUCH closer

🞎 FIRST statement is SOMEWHAT closer

🞎 SECOND statement is MUCH closer

🞎 SECOND statement is SOMEWHAT closer

🞎 Don’t know

🞎 REFUSED

# **Domain 2: Unintended Impacts**

Now, I’d like to ask a few questions on how the new tax on sugary drinks might affect people and businesses in Seattle.

- 1. Like I did earlier, I’m going to read you pairs of statements that people have made about this new tax on sugary drinks. After I read each pair, please tell me which statement is closer to your own view, even if neither is exactly right.

**(INTERVIEWER PROMPT)** Which statement comes closer to your own view?

| ____ 5A | **Statement 1:** I WILL travel to another city to buy sugary drinks so I don’t have to pay the tax.  **Statement 2:** I will NOT travel to another city to buy sugary drinks because of the tax. |
| --- | --- |
| ____ 5B | **Statement 1:** This tax will have a POSITIVE effect on Seattle's economy.  **Statement 2:** This tax will have a NEGATIVE effect on Seattle's economy. |
| ____ 5C | **Statement 1:** This tax WILL have a negative effect on small businesses in Seattle. Small businesses may lose money and could even go out of business because of the tax.  **Statement 2:** This tax will NOT have negative effects on small businesses in Seattle. It’s not likely that businesses will lose money or go out of business because of the tax. |
| ____ 5D | **Statement 1:**  This tax WILL result in job loss in Seattle.  **Statement 2:**  This tax will NOT result in job loss in Seattle. |
| ____ 5E | **Statement 1:** This tax WILL have a negative impact on my family's finances  **Statement 2:** This tax will NOT have a negative impact on my family's finances. |
| ____5F | **Statement 1:** This tax will have a POSITIVE impact on low-income and minority people’s health and well-being and help them access affordable, healthy food in Seattle.  **Statement 2:** This tax will have a NEGATIVE impact on low-income and minority people’s finances, will drive up the cost of living for those who can least afford to pay the tax, and further increase income inequality. |

**(AFTER CHOICE IS MADE, INTERVIEWER PROBE:)** Is that MUCH closer or SOMEWHAT closer?

🞎 FIRST statement is MUCH closer

🞎 FIRST statement is SOMEWHAT closer

🞎 SECOND statement is MUCH closer

🞎 SECOND statement is SOMEWHAT closer

🞎 Don’t know

🞎 REFUSED

- 1. Similar to prior questions, I'm going to read you a pair of statements. After I read both statements please tell me which one comes closer to your own view, even if neither is exactly right.

**(INTERVIEWER PROMPT)** Which statement comes closer to your own view?

**Statement 1:** Under this tax, people will still have the CHOICE to drink what they want.

**Statement 2:** This tax will significantly LIMIT people's ability to choose what they drink.

**(AFTER CHOICE IS MADE, INTERVIEWER PROBE:)** Is that MUCH closer or SOMEWHAT closer?

🞎 FIRST statement MUCH closer

🞎 FIRST statement SOMEWHAT closer

🞎 SECOND statement MUCH closer

🞎 SECOND statement SOMEWHAT closer

🞎 Don’t know

🞎 REFUSED

# **Domain 5: Conclusion and Demographics**

- 1. After hearing more about the tax, let me ask you again, do you strongly disapprove, somewhat disapprove, somewhat approve, or strongly approve of this tax?

🞎 Strongly disapprove

🞎 Somewhat disapprove

🞎 Somewhat approve

🞎 Strongly approve

🞎 Don’t know

🞎 REFUSED

Finally, I want to ask you a few questions about yourself and your household.

- 1. What is your age?

🞎 18-30

🞎 31-40

🞎 41-50

🞎 51-64

🞎 65+

🞎 REFUSED

- 1. What is your gender?

🞎 Male 🞎 Female 🞎 Self-identify(Specify:_______________ 🞎 REFUSED

- 1. What was your highest education level you completed?

🞎 Some high school

🞎 Completed high school

🞎 Some college or vocational training

🞎 Completed college or university

🞎 Completed graduate or professional degree

🞎 REFUSED

- 1. What is your marital status?

🞎 Married

🞎 Widowed/divorced/separated

🞎 Single and never married

🞎 Living with partner

🞎 REFUSED

- 1. Now, we don’t want to know your exact income, but just roughly, could you tell me if your annual household income before taxes is:

🞎 <$30,000

🞎 $30,000-$59,999

🞎 $60,000-$89,999

🞎 $90,000-$120,000

🞎 >$120,000

🞎 DON’T KNOW

🞎 REFUSED

- 1. Can you tell me if you have been covered by Medicaid in the last 12 months?

🞎 Yes

🞎 No

🞎 DON’T KNOW

🞎 REFUSED

- 1. Generally speaking, do you think of yourself as **(ROTATE)** a Democrat, an Independent, a Republican, or what?

🞎 Democrat

🞎 Independent

🞎 Republican

🞎 Other (SPECIFY)

🞎 DON’T KNOW

🞎 REFUSED

- 1. To help us make sure people from all Seattle neighborhoods are included in this survey, we would like to know the nearest intersection to your home. Please name the two cross-streets of this intersection.

What is the name of the first street? ___________________________

INTERVIEWER NOTE: Confirm street spelling and directionals (e.g. N, S, NW, NE)

What is the name of the second street?_________________________

INTERVIEWER NOTE: Confirm street spelling and directionals (e.g. N, S, NW, NE)

1. The web version of the survey was identical in content, but some questions were phrased differently because there was not an interviewer reading the questions. [↑](#footnote-ref-1)
